# Entropy-Regularized Iterative Weighted Shrinkage-Thresholding Algorithm (ERIWSTA) for inverse problems in imaging

**DOI:** 10.1371/journal.pone.0311227

**Published:** 2024-12-27

**Authors:** Limin Ma, Bingxue Wu, Yudong Yao, Yueyang Teng

**Affiliations:** 1 College of Medicine and Biological Information Engineering, Northeastern University, Shenyang, Liaoning Province, China; 2 Department of Electrical and Computer Engineering, Stevens Institute of Technology, Hoboken, NJ, United States of America; 3 Key Laboratory of Intelligent Computing in Medical Image, Ministry of Education, Shenyang, Liaoning Province, China; Shanghai University, CHINA

## Abstract

The iterative shrinkage-thresholding algorithm (ISTA) is a classic optimization algorithm for solving ill-posed linear inverse problems. Recently, this algorithm has continued to improve, and the iterative weighted shrinkage-thresholding algorithm (IWSTA) is one of the improved versions with a more evident advantage over the ISTA. It processes features with different weights, making different features have different contributions. However, the weights of the existing IWSTA do not conform to the usual definition of weights: their sum is not 1, and they are distributed over an extensive range. These problems may make it challenging to interpret and analyze the weights, leading to inaccurate solution results. Therefore, this paper proposes a new IWSTA, namely, the entropy-regularized IWSTA (ERIWSTA), with weights that are easy to calculate and interpret. The weights automatically fall within the range of [0, 1] and are guaranteed to sum to 1. At this point, considering the weights as the probabilities of the contributions of different attributes to the model can enhance the interpretation ability of the algorithm. Specifically, we add an entropy regularization term to the objective function of the problem model and then use the Lagrange multiplier method to solve the weights. Experimental results of a computed tomography (CT) image reconstruction task show that the ERIWSTA outperforms the existing methods in terms of convergence speed and recovery accuracy.

## Introduction

Inverse problems in imaging are essential for physical and biomedical sciences [1]. They have been widely used in optical and radar systems, including X-ray computed tomography (CT), positron emission tomography (PET), and electrical tomography (ET). An inverse problem in imaging aims to estimate an unknown image from the given measurements by solving an optimization problem with a regularizer. Mathematically, it is to infer an original signal *x* ∈ *R*^*n*^ from its measurements *b* = *Ax* ∈ *R*^*m*^. Here, *A* ∈ *R*^*m* × *n*^ is a linear random projection (matrix). Because *m* ≪ *n*, this inverse problem is typically ill-posed.

Therefore, researchers typically construct an optimization problem and utilize a regularizer to solve it. However, the employed regularizer is usually nonsmooth, so it cannot be solved in a straightforward manner. Therefore, many first-order iterative proximal gradient methods have also been proposed, e.g., the iterative shrinkage-thresholding algorithm (ISTA) [2], two-step ISTA (TwISTA) [3] and fast ISTA (FISTA) [4, 5]. The ISTA is one of the most popular methods for solving such problems, and its advantage is its simplicity. However, the ISTA has also been recognized as a slow method. TWIST, as a faster method than the ISTA, also has good effectiveness. The FISTA has low complexity, fast convergence, and moderate recovery accuracy. It has been proven that the FISTA can also be faster than TWIST by several orders of magnitude. In addition, the alternating direction method of multipliers (ADMM) [6] and primal-dual hybrid gradient (PDHG) algorithm [7] are important methods. These methods solve inverse problems with nonsmooth regularizers that possess high computational efficiency. Unfortunately, these methods treat every attribute equally, which can result in inaccurate optimal solutions.

Recently, the iterative weighted shrinkage-thresholding algorithm (IWSTA) [8] and its variants have been developed to extend the range of practical applications. These methods often outperform unweighted algorithms. Nasser *et al.* [9] proposed the weighted FISTA (W-FISTA), which has higher estimation efficiency than the original FISTA but the same complexity. Candes *et al.* [10] proposed an iterative algorithm for reweighted *L*_1_ minimization (IRL1) to penalize better nonzero coefficients. This method solves the imbalance between the *L*_1_ and *L*_0_ norms. Chartrand *et al.* [11] proposed an iterative reweighted least-squares (IRLS) algorithm to attain an improved ability to recover sparse signals. IRLS and IRL1 are known for their state-of-the-art reconstruction rates for noiseless and noisy measurements. Foucart *et al.* [12] proposed a weighted method to obtain the solution of a system with the minimal *L*_*q*_ quasinorm (WLQ). This method generalizes and improves the result obtained with the *L*_1_ norm. Wipf *et al.* [13] presented distinct detail-separable and nonseparable iterative reweighting algorithms and introduced mainly nonnegative sparse coding examples via reweighted *L*_1_ minimization (IRW) for solving linear inverse problems. However, the weights of these algorithms do not conform to the usual weight definition. Their sums are not one, and these weights are distributed over an enormous range. Such weights are difficult to explain and may lead to inaccurate results. Table 1 lists some IWTAs and their details.

**Table 1:** Variants of weighted methods.

Algorithm	Reference	Weights	Min.	Max.	Regularizer
ISTA	Daubechies *et al.* [2]	1	1	1	$\sum_{i=1}^{n}\left|x_{i}\right|$
IRL1	Candes *et al.* [10]	$\frac{1}{|x_{i}^{k-1}|+\delta }$	0	$\frac{1}{\delta }$	$\sum_{i=1}^{n}\text{log}(|x_{i}\left|+\delta \right)$
WLQ	Foucart *et al.* [12]	$\frac{1}{{\left(|x_{i}^{k-1}|+\delta \right)}^{1-p}}$	0	$\frac{1}{\delta^{1-p}}$	$\frac{1}{p}\sum_{i=1}^{n}\left(\right|x_{i}{\left|+\delta \right)}^{p}$
IRW	Wipf *et al.* [13]	$\frac{1+\left(\right|x_{i}{\left|+\delta \right)}^{p+1}}{\left(\right|x_{i}{\left|+\delta \right)}^{p+1}}$	0	1	$\sum_{i=1}^{n}\left(\right|x_{i}\left|-\frac{1}{{\left(x_{i}+\delta \right)}^{p}}\right)$

This paper proposes a new IWSTA based on entropy regularization called the entropy-regularized IWSTA (ERIWSTA). This algorithm makes the weights easy to calculate, and it has good interpretability. Additionally, the iterative formula update process becomes simple. Experimental results obtained in synthetic CT image denoising tasks show that the proposed method is feasible and effective.

## Related work

We introduce the general model of an inverse problem and its solving method (the ISTA). For the convenience of description, we present the symbolic notations. Matrices are represented as capital letters. For a matrix *A*, A_*i_, *A*_i*_ and *A*_*ij*_ denote the *i*-th column, the *i*-th row and the (*i*, *j*)-th element of *A*, respectively; ‖ ⋅ ‖_*i*_ represents the *i*-norm of a vector. All the vectors are column vectors unless transposed to a row vector by a prime superscript *T*.

The equation of an inverse problem in imaging can be expressed as follows [14]:

$$\begin{array}{c} b=Ax+\epsilon , \end{array}$$ (1)

where *x* ∈ *R*^*n*^ denotes an unknown image, *b* ∈ *R*^*m*^ denotes the given measurements, *A* ∈ *R*^*m* × *n*^ is called the system matrix (usually, *m* ≪*n*), and *ϵ* is the unknown disturbance term or noise. Note that the system is underdetermined. To reconstruct the original image *x*, researchers usually construct the following optimization problem [15]:

$$\begin{array}{c} \underset{x}{\text{min}}\frac{1}{2}{∥Ax-b∥}_{2}^{2}. \end{array}$$ (2)

Generally, the least-squares method is used to solve the problem.

To suppress overfitting, some scholars [16–18] have introduced the *L*_0_ norm to Eq (2) as sparse prior information. Therefore, to obtain the solution of Eq (2), we must solve an optimization problem that minimizes the cost function [19]:

$$\begin{array}{c} \underset{x}{\text{min}}\frac{1}{2}{∥Ax-b∥}_{2}^{2}+\beta {∥x∥}_{0}, \end{array}$$ (3)

where ‖*x*‖_0_, the number of nonzero components of *x*, is the regularizer that imposes prior knowledge (sparsity); *β* > 0, the regularization coefficient, is a hyperparameter used to control the tradeoff between accuracy and sparsity. Eq (3) is an NP-hard optimization problem [20], which is highly discrete, so it is challenging to precisely solve this problem. Thus, we must seek an effective approximation solution for this problem. The *L*_1_ norm regularizer is introduced as a substitute for the *L*_0_ norm. That is [2],

$$\begin{array}{c} \underset{x}{\text{min}}\frac{1}{2}{∥Ax-b∥}_{2}^{2}+\beta {∥x∥}_{1}. \end{array}$$ (4)

This is a convex continuous optimization problem with a sole nondifferentiable point (*x* = 0). The classic method for solving the problem is the ISTA proposed by Chambolle *et al.* [21, 22]. The ISTA updates *x* through the following shrinkage and soft thresholding operation in each iteration:

$$\begin{array}{c} x^{k+1}=soft_{\beta t}\left[x^{k}-2tA^{T}\left(Ax^{k}-b\right)\right], \end{array}$$ (5)

where *k* represents the *k*-th iteration, *t* is an appropriate step size and *soft* is the soft threshold operation function. The *soft* function has the following form:

$$\begin{array}{c} soft_{\theta }\left(x_{i}\right)=sign\left(x_{i}\right)\left(\right|x_{i}\left|-\theta \right), \end{array}$$ (6)

where *sign*(*x*_*i*_) is the sign function of *x*_*i*_ and *θ* is the threshold.

Recently, the IWSTA has attracted more interest than the ISTA, as it outperforms its unweighted counterparts in most cases. In these methods, decision variables and weights are optimized in an alternating manner, or decision variables are optimized under heuristically chosen weights. Usually, the objective function of this type of algorithm has the following form [13]:

$$\begin{array}{c} \underset{x,\;w\geq 0}{\text{min}}\frac{1}{2}{∥Ax-b∥}_{2}^{2}+\beta \sum_{i=1}^{n}w_{i}{\left|x_{i}\right|}_{1}. \end{array}$$ (7)

In this paper, we improve the second term in Eq (7) to obtain a variant. Then, we propose an iterative update law for the variant.

## Methodology

The main idea of IWSTA-type algorithms is to define a weight for each attribute based on the current iteration *x*^*k*^ and then use the defined weights to obtain a new *x*. In this section, we introduce an entropy regularizer to the cost function and obtain the following optimization model:

$$\begin{array}{ccc} \text{min} & & \Phi_{\beta ,\gamma }\left(x,w\right)=F\left(x\right)+\beta G_{\gamma }\left(x,w\right)\\ s.\;t. & & w_{i}\geq 0,\;\sum_{i=1}^{n}w_{i}=1\\ where & & F\left(x\right)=\frac{1}{2}{∥Ax-b∥}_{2}^{2}\\ & & G_{\gamma }\left(x,w\right)=\sum_{i=1}^{n}w_{i}\left|x_{i}\right|+\gamma \sum_{i=1}^{n}w_{i}\;\text{ln}\;w_{i} \end{array}$$ (8)

where *γ* ≥ 0 is a given hyperparameter, and the inspiration for adding a term with logarithms comes from the literature [23]. It is worth noting that if we do not use the entropy regularizer, *w* can easily be solved as *w*_*i*_ = 1 when |*x*_*i*_| = min{|*x*_1_|, …, |*x*_*n*_|}, and otherwise is 0 The update rule can be easily explained by an example as

$$\begin{array}{ll} min\;\{4,1,5\}= & min\;4w_{1}+1w_{2}+5w_{3}\\ & s.\;t.\;w_{1},w_{2},w_{3}\geq 0\\ & w_{1}+w_{2}+w_{3}=1 \end{array}$$

The solutions are *w*_1_ = 0, *w*_2_ = 1 and *w*_3_ = 0, among which *w*_2_ corresponds to the minimum value of {4, 1, 5}. This is very similar to the weight computation in the k-means algorithm. This shows the simple fact that only one element of *w* is 1, and the others are 0, which is grossly incompatible with the actual problem. Then, we add the negative entropy of the weights to measure the uncertainty of the weights and stimulate more attributes to help with signal reconstruction because it is well known that $\sum_{i=1}^{n}w_{i}\;\text{ln}\;w_{i}$ is minimized in information theory when

$$\begin{array}{c} w_{1}=w_{2}=...=w_{n} \end{array}$$ (9)

We alternatively solve *w* and *x* in Eq (8) as follows.

### Update rule for *w*

To solve *w*, we introduce the Lagrange multiplier λ and obtain the following Lagrange function. For *w*, *F*(*x*) is a constant, so we only construct a Lagrange function on *G*(*x*), which can be expressed as follows:

$$\begin{array}{c} L\left(w,\lambda \right)=G_{\gamma }\left(x,w\right)+\lambda \left(\sum_{i=1}^{n}w_{i}-1\right). \end{array}$$ (10)

We set the partial derivatives of *L*(*w*, λ) with respect to *w*_*i*_ and λ to zero and then obtain the following two equations:

$$\begin{array}{ccc} \frac{\partial L(w,\lambda )}{\partial w_{i}} & = & |x_{i}|+\gamma \left(1+\;\text{ln}\;w_{i}\right)+\lambda =0, \end{array}$$ (11)

$$\begin{array}{ccc} \frac{\partial L(w,\lambda )}{\partial \lambda } & = & \sum_{i=1}^{n}w_{i}-1=0. \end{array}$$ (12)

From Eq (11), we know that

$$\begin{array}{c} w_{i}=\text{exp}\left(-\frac{\lambda }{\gamma }\right)\text{exp}\left(-\frac{|x_{i}|}{\gamma }\right)\text{exp}\left(-1\right). \end{array}$$ (13)

Substituting Eq (13) into Eq (12), we have

$$\begin{array}{c} \sum_{i=1}^{n}w_{i}=\text{exp}\left(-\frac{\lambda }{\gamma }\right)\sum_{i=1}^{n}\text{exp}\left(-\frac{|x_{i}|}{\gamma }\right)\text{exp}\left(-1\right)=1. \end{array}$$ (14)

It follows that

$$\begin{array}{c} \text{exp}\left(-\frac{\lambda }{\gamma }\right)=\frac{{\text{exp}}^{1}}{\sum_{i=1}^{n}\text{exp}\left(-\frac{|x_{i}|}{\gamma }\right)}. \end{array}$$ (15)

Substituting this expression into Eq (13), we obtain that

$$\begin{array}{c} w_{i}=\frac{\text{exp}(-\frac{|x_{i}|}{\gamma }+1)}{\sum_{l=1}^{n}\text{exp}\left(-\frac{|x_{l}|}{\gamma }\right)}. \end{array}$$ (16)

Such weights certainly satisfy the constraints that *w*_*i*_ ≥ 0 and $\sum_{i=1}^{n}w_{i}=1$.

### Update rule for *x*

Inspired by the work concerning the ISTA, we adopt a similar approach for the iterative update process of *x*. The construction of a majorization is an important step toward obtaining the update rule.

**Definition 0.1** *(Majorization) Assume* Ψ(*x*) *is an n-dimensional real valued function about vector x*, *we can denote ψ*^*k*^(*x*;*x*^*k*^) *as a majorization for* Ψ(*x*) at *x*^*k*^ *(fixed) if ψ*^*k*^(*x*^*k*^) = Ψ(*x*^*k*^) *and ψ*^*k*^(*x*) ≥ Ψ(*x*).

Clearly, Ψ(*x*) is nonincreasing under the update rule *x*^*k*+1^ = min_*x*_*ψ*(*x*|*x*^*k*^) because

$$\begin{array}{c} \Psi \left(x^{k+1}\right)\leq \psi \left(x^{k+1}|x^{k}\right)\leq \psi \left(x^{k}|x^{k}\right)=\Psi \left(x^{k}\right). \end{array}$$ (17)

Then, we can construct a majorization for *F*(*x*).

**Remark 0.1**. *Conditions for constructing surrogate function: A basic principle of optimization algorithm is to construct an easily minimized function ψ*^*k*^(*x*;*x*^*k*^) *to replace the original function* Ψ(*x*). *Then calculate the minimum value of the function ψ*^*k*^(*x*;*x*^*k*^), *and use the minimum value point as the new iteration point(i.e. x*^*k*+1^ = *argmin ψ*^*k*^(*x*;*x*^*k*^)). *By continuously repeating the two steps of constructing the surrogate function and finding the minimum value of the surrogate function, an estimated sequence ψ*^*k*^ *of* Ψ *can be obtained. The estimated sequence ψ*^*k*^ *makes* Ψ(*x*^*k*^) *monotonically decreasing when the surrogate function satisfies the following conditions* [24, 25]:

$$\begin{array}{c} \psi^{k}\left(x^{k}\right)=\Psi \left(x^{k}\right) \end{array}$$ (18a)

$$\begin{array}{c} \psi^{k}\left(x\right)\geq \Psi \left(x\right),x\in D \end{array}$$ (18b)

**Proposition 0.1** *Notably, F*(*x*) *is a Lipschitz-continuous and differentiable convex function, which has a majorization function at the fixed current iteration x*^*k*^ *as follows:*

$$\begin{array}{c} f\left(x,x^{k}\right)=F\left(x^{k}\right)+{\left[\nabla F\left(x^{k}\right)\right]}^{T}\left(x-x^{k}\right)+\frac{L}{2}{∥x-x^{k}∥}_{2}^{2}, \end{array}$$ (19)

*where L is larger than or equal to the maximum eigenvalue of A*^*T*^*A*.

*Proof*. It is well known that

$$\begin{array}{c} F\left(x\right)=\frac{1}{2}{∥Ax-b∥}_{2}^{2}=F\left(x^{k}\right)+{\left[\nabla F\left(x^{k}\right)\right]}^{T}\left(x-x^{k}\right)+\frac{1}{2}{\left(x-x^{k}\right)}^{T}A^{T}A\left(x-x^{k}\right) \end{array}$$ (20)

Comparing *F*(*x*) with *F*(*x*, *x*^*k*^), we find that their last items are different. By performing singular value decomposition (SVD) on a symmetric definite matrix, we know that *A*^*T*^*A* = *Q*^*T*^*ΣQ*, in which *Q* is an orthogonal matrix consisting of all eigenvectors and *Σ* is a diagonal consisting of all eigenvalues. Let *z* = *x* − *x*^*k*^; then,

$$\begin{array}{c} z^{T}\left(A^{T}A\right)z=z^{T}Q^{T}\Sigma Qz\leq {L∥Qz∥}_{2}^{2}=L{∥z∥}_{2}^{2} \end{array}$$ (21)

It is also certain that $z^{T}A^{T}Az=L{∥z∥}_{2}^{2}=0$ if *x* = *x*^*k*^. Thus, the proof is established. Now, we obtain the majorization for the cost function Φ(*x*, *w*) on *x*.

$$\begin{array}{c} \phi \left(x,x^{k}\right)=f\left(x,x^{k}\right)+\beta G_{\gamma }\left(x,w\right), \end{array}$$ (22)

which can be reorganized as:

$$\begin{array}{ccc} \phi (x,x^{k}) & = & \frac{L}{2}{∥x-[x^{k}-\frac{1}{L}\nabla F\left(x^{k}\right)]∥}_{2}^{2}+\beta G_{\gamma }\left(x,w\right)\\ & = & \sum_{i=1}^{n}\{\frac{L}{2}∥x_{i}-{\left[x^{k}-\frac{1}{L}\nabla F\left(x^{k}\right)\right]}_{i}{∥}_{2}^{2}+\beta w_{i}|x_{i}\left|\right\}+constant. \end{array}$$ (23)

Then,

$$\begin{array}{c} x^{k+1}=argmin\sum_{i=1}^{n}\frac{L}{2}{\left\{x_{i}-{\left[x^{k}-\frac{1}{L}\nabla F\left(x^{k}\right)\right]}_{i}\right\}}_{2}^{2}+\beta w_{i}\left|x_{i}\right|. \end{array}$$ (24)

where (.)_*i*_ denotes the *i*-th element of vector (.).

Let

$$\begin{array}{c} r^{k}:=x^{k}-\frac{1}{L}\nabla F\left(x^{k}\right), \end{array}$$ (25)

so we know that

$$\begin{array}{c} x^{k+1}=argmin\sum_{i=1}^{n}\frac{L}{2}{\left(x_{i}-r_{i}^{k}\right)}^{2}+\beta w_{i}\left|x_{i}\right|. \end{array}$$ (26)

Let

$$\begin{array}{c} g\left(x_{i}\right)=\frac{L}{2}{\left(x_{i}-r_{i}^{k}\right)}^{2}+\beta w_{i}\left|x_{i}\right|. \end{array}$$ (27)

Let $x_{i}^{k}$ be the value of *x*_*i*_ when the partial derivative $\frac{\partial g(x_{i})}{\partial x_{i}}$ is 0, that is:

$$\begin{array}{c} \frac{\partial g(x_{i})}{\partial x_{i}}=L\left(x_{i}^{k}-r_{i}^{k}\right)+\beta w_{i}sign\left(x_{i}^{k}\right)=0, \end{array}$$ (28)

By transferring terms, we can obtain the following equation:

$$\begin{array}{c} r^{k}=x_{i}^{k}+\frac{1}{L}\beta w_{i}sign\left(x_{i}^{k}\right) \end{array}$$ (29)

That is,

$$\begin{array}{c} x_{i}^{\left(k+1\right)}=x_{i}^{k}+\frac{1}{L}\Delta F\left(x_{i}^{k}\right)+\frac{1}{L}\beta w_{i}sign\left(x_{i}^{k}\right) \end{array}$$ (30)

Due to $t=\frac{1}{L}$ and *ΔF*(*x*) = 2*A*^*T*^(*Ax* − *b*), the iterative formula can be easily obtained as follows:

$$\begin{array}{c} x_{i}^{k+1}=soft_{\beta tw_{i}}\left(r_{i}^{k}\right)=soft_{\beta tw_{i}}\left[x_{i}^{k}-2tA^{T}\left(Ax_{i}^{k}-b\right)\right] \end{array}$$ (31)

Therefore, the update of *x* is completed.

## Experiments

### Experimental description

**Fig 1:**
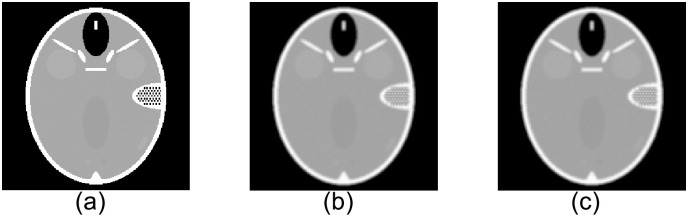
Original and noisy head phantom images. (*a*) Head phantom with 256 × 256 pixels; (*b*) and (*c*) blurred images with 5 × 5 uniform kernels and additive Gaussian noise with variances of *σ* = 10^−2^ and *σ* = 10^−3^, respectively.

We use a simulated CT dataset and a real PET dataset to evaluate the performance of the ERWISTA. The simulated CT dataset consists of a Shepp-Logan phantom with 256 × 256 pixels. The use of phantom data brings many advantages, including the fact that we have accurate a priori knowledge of the pixel values and the freedom to add noise to them as needed. We blur the image (*b*) using a uniform 5 × 5 kernel (applied by the fspecial MATLAB function) and then add Gaussian noise with a mean of 0 and variances of 10^−2^ and 10^−3^. The original and blurred and noisy images are shown in Fig 1. The real dataset comes from the cooperating hospital. The dataset includes low-dose and normal-dose PET images with size of 256 × 256. All experiments are performed on an HP computer with a 2.5 GHz Intel(R) Core(TM) i7–4710MQ CPU with 12 GB of memory using MATLAB R2019a for coding.

### Evaluation standard

This paper uses the mean absolute error (MAE) to measure the similarity between the reconstructed and true images. The value of the MAE is calculated by taking the average of the squared differences between the restored pixel values and the true pixel values. Let *x* represent the ground truth, $\hat{x}$ represent the reconstructed image and *N* denote the number of voxels. The MAE is defined as follows:

$$\begin{array}{c} MAE=\frac{1}{N}||x-\hat{x}{\left|\right|}_{1}. \end{array}$$ (32)

Generally, lower MAE values indicate better reconstructed image quality.

### Experimental results of simulated dataset

**Fig 2:**
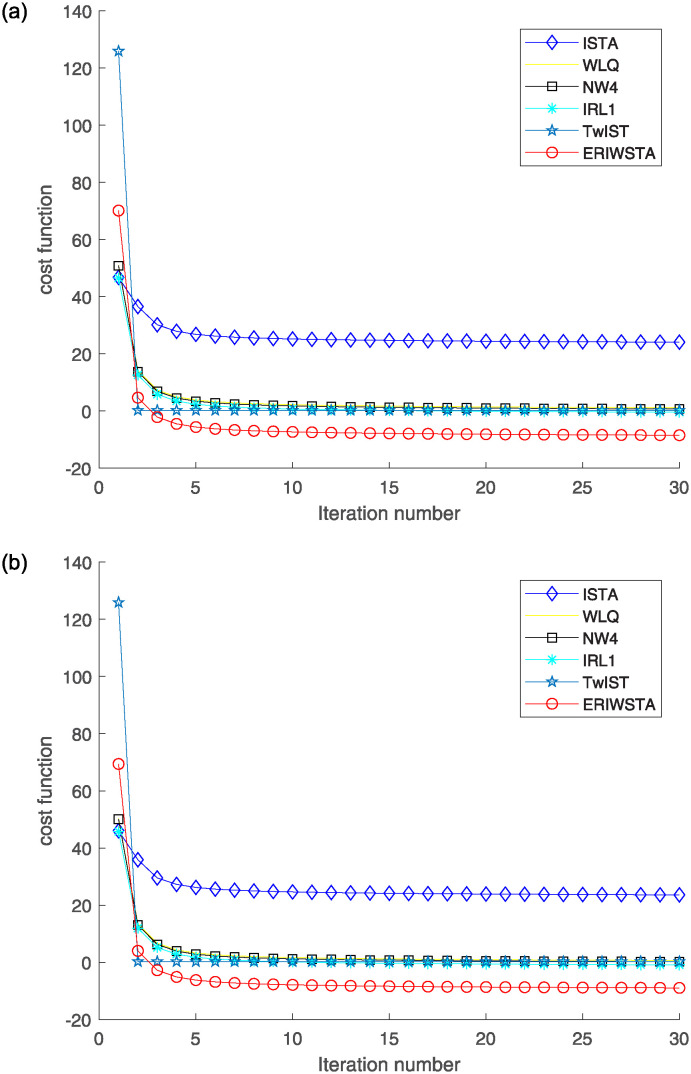
The cost function Φ(*x*, *w*) versus the number of iterations for different Gaussian noise levels: (a) *σ* = 10^−2^ and (b) *σ* = 10^−3^.

To illustrate the performance of the proposed method, we provide some visual results. Fig 2 displays the cost function curves produced by six algorithms during training. They are the ISTA, WLQ, IRW, IRL1, TwIST and the ERIWSTA. As is well known, we should not compare the values of different cost functions. However, we can visually compare their convergence speeds, in which an algorithm shows faster convergence when the corresponding curve becomes flatter within fewer iterations. We find that the six algorithms tend to converge after approximately several iterations and that the proposed algorithm has a fast convergence speed. The ERIWSTA arrives at the stable status early.

**Fig 3:**
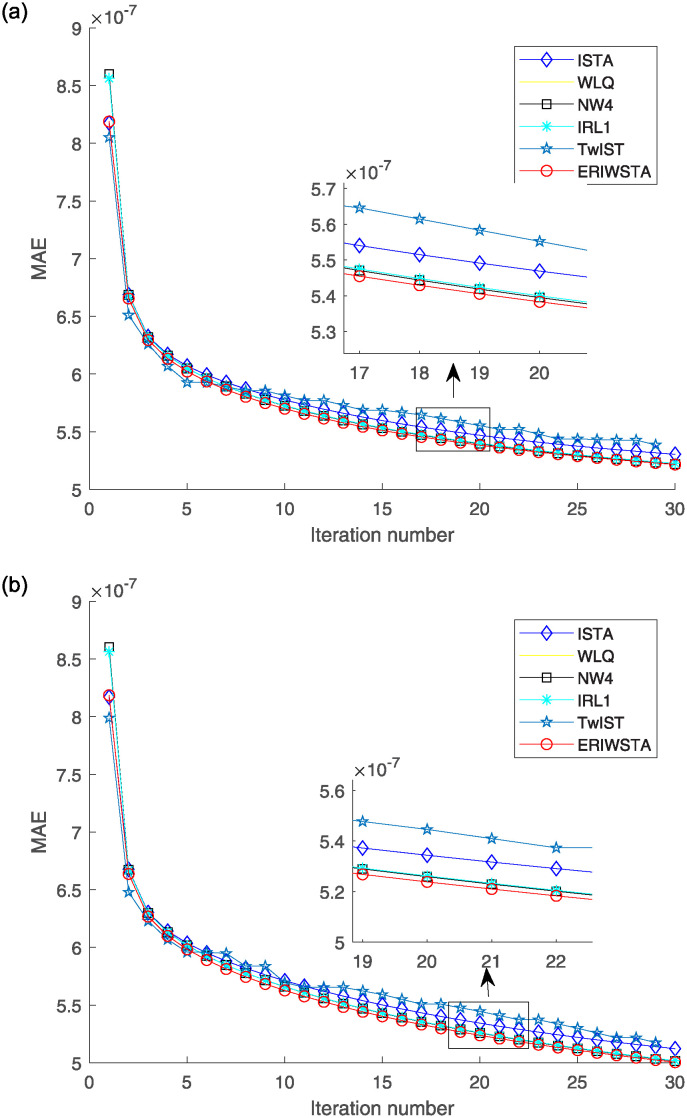
MAE versus the number of iterations for different Gaussian noise levels: (a) *σ* = 10^−2^ and (b) *σ* = 10^−3^.

Fig 3 shows the MAE curves of the six algorithms versus the number of iterations. Subgraphs (a) and (b) denote the curves produced in the cases with Gaussian noise levels of *σ* = 10^−2^ and *σ* = 10^−3^, respectively. We can observe that each algorithm has a small MAE value, indicating that all methods have considerable denoising capabilities. However, compared with the other algorithms, the ERIWSTA obtains the minimum MAE value after each iteration to obtain a clearer denoised image by observing an enlarged detail image.

**Fig 4:**
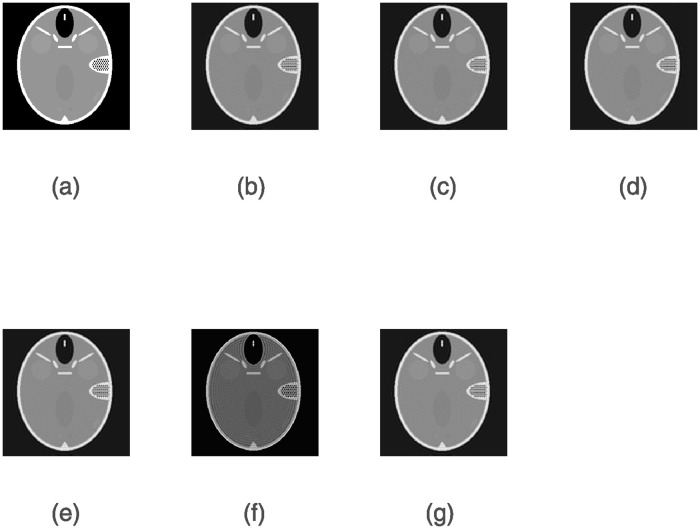
The denoising results yielded by different algorithms on a dataset with Gaussian noise at a level of *σ* = 10^−2^ after 30 iterations. (a) denotes the original image, and (b)–(g) denote the denoised images produced by the ISTA, WLQ, IRW, IRL1, TwIST and the ERIWSTA, respectively.

**Fig 5:**
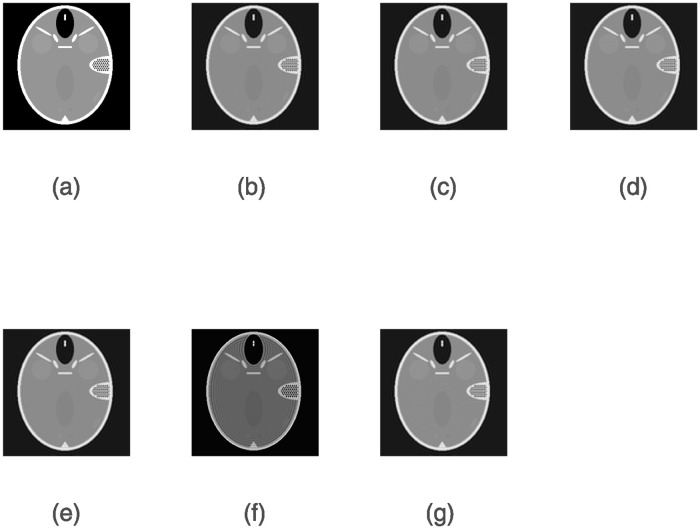
The denoising results yielded by different algorithms on a dataset with Gaussian noise at a level of *σ* = 10^−3^ after 30 iterations. (a) denotes the original image, and (b)-(g) denote the denoised images produced by the ISTA, WLQ, IRW, IRL1, TwIST and the ERIWSTA, respectively.

Figs 4 and 5 show the denoising results obtained by six algorithms under the given noise level. As seen, the image reconstructed by the ERIWSTA has a better noise removal effect than those of the other algorithms, and it can more accurately recover the edge information and texture features of brain images.

**Fig 6:**
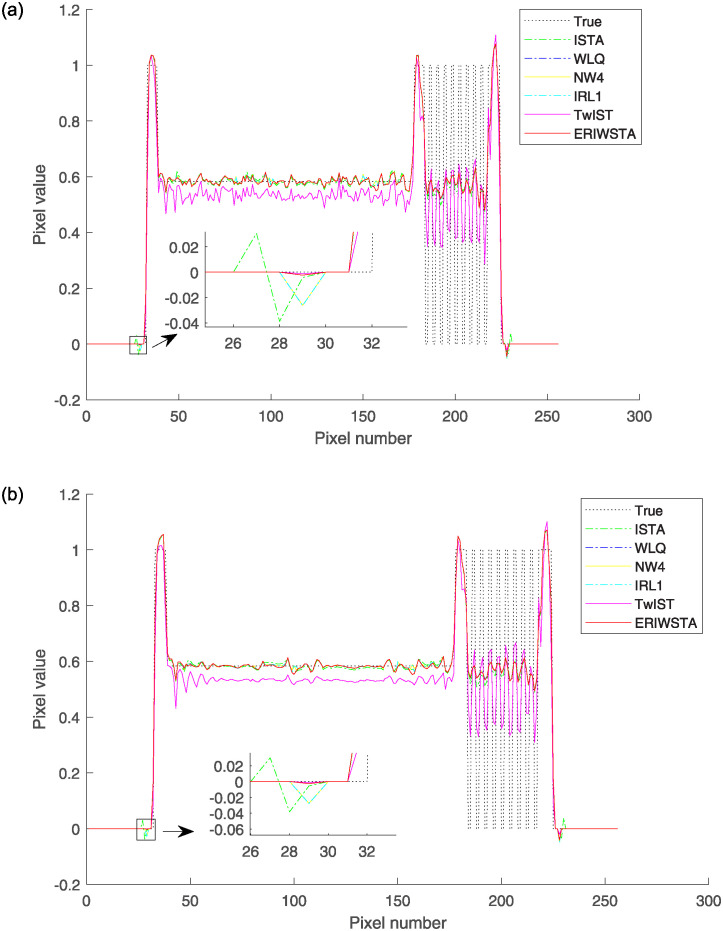
Horizontal central profiles produced for the 128-th row of the restored images with different Gaussian noise levels: (a) *σ* = 10^−2^ and (b) *σ* = 10^−3^.

**Fig 7:**
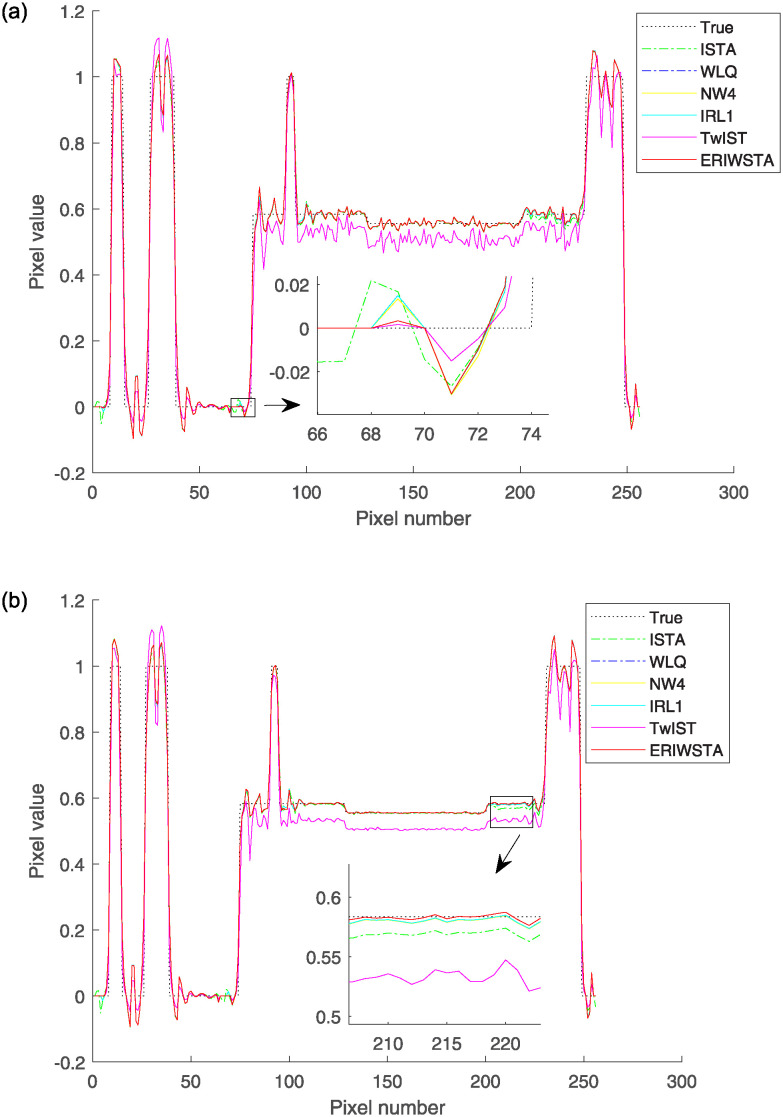
Vertical central profiles produced for the 128-th column of the restored images with different Gaussian noise levels: (a) *σ* = 10^−2^ and (b) *σ* = 10^−3^.

In addition, we further verify the effectiveness of the proposed algorithm. We select the same row and column of four reconstructed images for one slice and compare them with the corresponding rows and columns of the real image by their pixel values. Figs 6 and 7 show the comparisons among the horizontal and vertical center profiles of the restored images, respectively. IRL1 yields significant deviations from the true values in both the horizontal and vertical regions. The ISTA, IRW, WLQ and TwIST recover well in the horizontal region but sometimes have abnormal states in the vertical region. These methods cannot guarantee a stable denoising effect. Compared with the other algorithms, the ERIWSTA does not guarantee the lowest denoising errors in all intervals, but it can fit the true values more accurately overall. The ERIWSTA also has the best stability and robustness, can guarantee good denoising effects in areas with minor pixel value variations, and can track the true profile more accurately.

#### Hyperparameter selection

**Fig 8:**
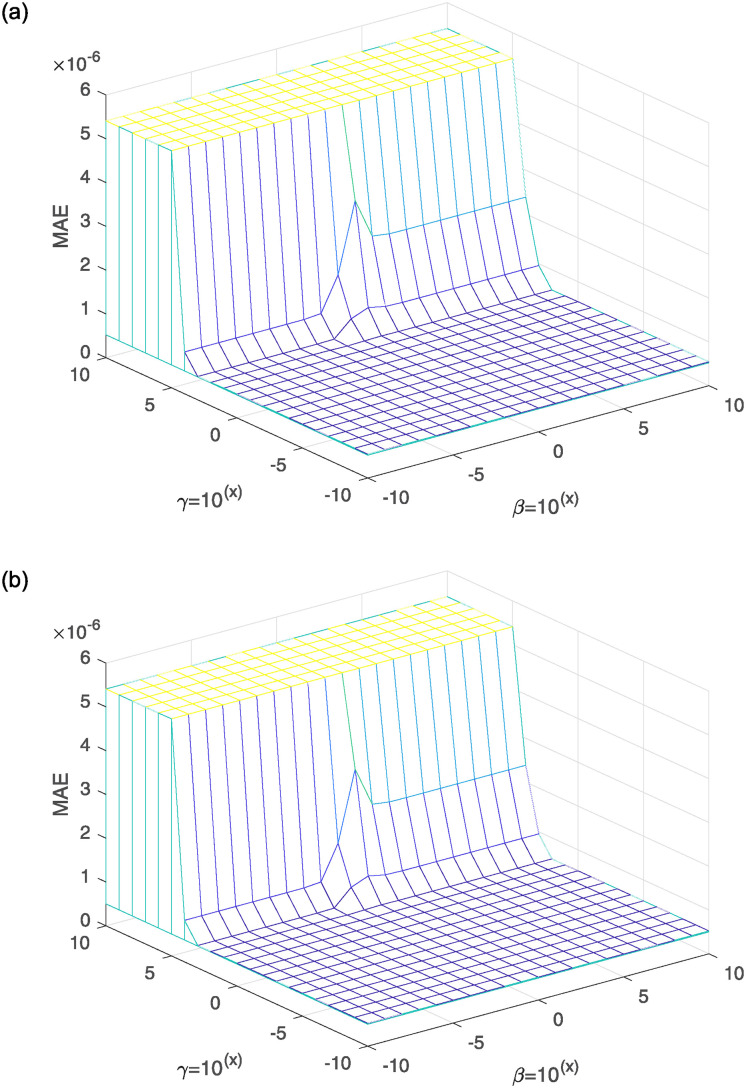
3D profiles of *β* and λ with respect to the MAE for different Gaussian noise levels: (a) *σ* = 10^−2^ and (b) *σ* = 10^−3^.

We conduct hyperparameter experiments to select the optimal penalty hyperparameter *β* and the optimal entropy-weighted hyperparameter *γ*. We provide a parameter range from 10^−10^ to 10^10^ and choose the MAE as the evaluation index. The number of experimental iterations is 100, and we plot a three-dimensional line graph for the ERIWSTA with the vertical axis representing the MAE values corresponding to different hyperparameters, as shown in Fig 8. The optimal values of *β* and *γ* can be chosen from wide ranges, and the ERIWSTA has stable lower MAE values for both, which also shows the excellent robustness of the ERIWSTA. In addition, to ensure the validity of the experimental results, we choose the optimal *β* and *γ* values for all the compared algorithms. For details, Tables 2 and 3 show the optimal values of all the algorithms’ *β* and *γ* parameters when conducting experiments on the datasets with noise levels of *σ* = 10^−2^ and *σ* = 10^−3^, respectively. An interesting observation is that, regardless of whether low or high noise levels are used, the restoration accuracy of our algorithm is always better than that of the other approaches.

**Table 2:** The optimal MAE values and corresponding hyperparameters (Gaussian noise with *σ* = 10^−2^).

Termed	*β*	*γ*	*δ*	MAE
**ISTA**	10^−3^	−	−	5.312077*10^−7^
**WLQ**	10^−5^	10^−10^	10^−3^	5.228672 * 10^−7^
**IRW**	10^−5^	10^−2^	10^−3^	5.410231 * 10^−7^
**IRL1**	10^−5^	−	10^−3^	5.228672 * 10^−7^
**TwIST**	10^−5^	−	10^−5^	5.333443 * 10^−7^
**ERIWSTA**	10^2^	10^−2^	−	5.218246 * 10^−7^

**Table 3:** The optimal MAE values and corresponding hyperparameters (Gaussian noise with *σ* = 10^−3^).

Termed	*β*	*γ*	*δ*	MAE
**ISTA**	10^−3^	−	−	5.122013 * 10^−7^
**WLQ**	10^−5^	10^−5^	10^−3^	5.018339 * 10^−7^
**IRW**	10^−5^	10^−2^	10^−3^	5.410231 * 10^−7^
**IRL1**	10^−5^	−	10^−3^	5.018340 * 10^−7^
**TwIST**	10^−5^	−	10^−5^	5.311041 * 10^−7^
**ERIWSTA**	10^2^	10^−2^	−	5.005524 * 10^−7^

### Experimental results of real PET dataset

**Fig 9:**
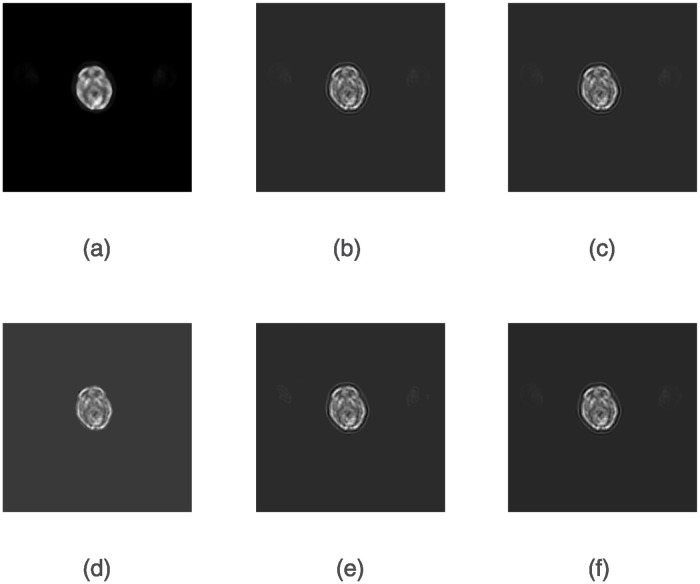
The denoising results yielded by different algorithms. (a) denotes the ground truth, and (b)–(f) denote the denoised images produced by the ISTA, WLQ, IRW, IRL1 and the ERIWSTA, respectively.

**Fig 10:**
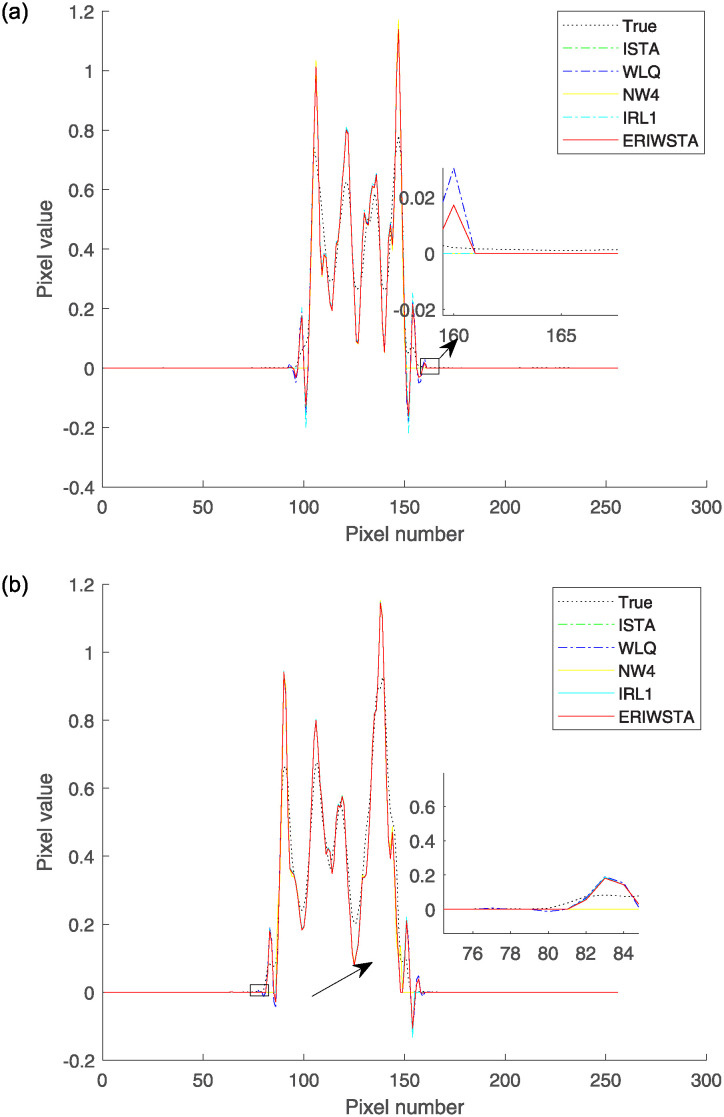
The comparison between PET images reconstructed by different algorithms and the label images focuses on specific pixel profiles: (a) shows the pixel values along the 128th row of the corresponding images. (b) shows the pixel values along the 128th column of the corresponding images.

To make the results more convincing, we added the experiment result of real PET image. Fig 9 shows the denoised images and the second to sixth columns of Fig 9 are the images reconstructed using ISTA, WLQ, IRW, IRL1 and the proposed method, respectively. It is seen that all methods have considerable denoising ability, and the proposed algorithm has a better ability to denoise. Fig 10 shows the comparisons among the horizontal and vertical center profiles of the restored PET images, respectively. The ISTA, IRW and WLQ recover well in the horizontal region but sometimes have abnormal states in the vertical region. Compared with the other algorithms, the ERIWSTA does not guarantee the lowest denoising errors in all intervals, but it can fit the true values more accurately overall. The ERIWSTA also has the best stability and robustness.

## Conclusions

This paper proposes a new IWSTA for solving linear inverse problems based on entropy regularization. The main innovation of the algorithm is its introduction of an entropy regularization term in the loss function of the classic ISTA. The weights of the new algorithm are easy to calculate and have good interpretability. In addition, the iterative process of updating the formula becomes simple. Finally, we demonstrate the algorithm’s effectiveness in CT and PET data denoising experiments.

Although the experimental results show that the proposed method effectively removes noise, this work still needs to be improved. The hyperparameters of all algorithms in this paper are obtained by manually adjusting them according to the resulting imaging quality. This selection method requires cyclic experiments for the selected parameter intervals to choose the optimal parameters. This method could be more efficient and select only the optimal parameters in the given interval. Therefore, in the future, we will study a method for adaptively setting the parameters to accurately select the optimal parameters of each algorithm, thus ensuring the algorithm’s effectiveness and further improving its noise reduction ability. In addition, the data used in this experiment are phantom data. In algorithmic analyses, the use of simulated data provides good properties. It is easy to add noise and is conducive to the theoretical study of an algorithm’s effectiveness. However, the proposed algorithm still must be verified in more practice. Therefore, verifying the algorithm’s effectiveness on more patients’ clinical data and effectively improving the algorithm based on an actual application would be meaningful.

## Supporting information

S1 Graphical abstract (PDF)
