# Supplementary material for: Entropy-Regularized Iterative Weighted Shrinkage-Thresholding Algorithm (ERIWSTA) for inverse problems in imaging
Source: PLoS One. 2024 Dec 27;19(12):e0311227. doi: 10.1371/journal.pone.0311227 (PMC11676579; doi:10.1371/journal.pone.0311227)

How to solve a linear inverse problem?

The classical approach
ISTA, WLP, NW1, IRL1

Disadvantage:The weight design of these algorithms needs to be improved.

The proposed method (ERIWSTA)

$$min \quad \emptyset_{\beta,\gamma}(x,w) = F(x) + \beta G_{\gamma}(x,w)$$

$$s.t. \quad \omega_i \geq 0, \sum_{i=1}^{n} w_i = 1$$

$$Where \quad F(X) = \frac{1}{2}||Ax - b||_2^2$$

$$G_{\gamma}(x,w) = \sum_{i=1}^{n} w_i |x_i| + \gamma \sum_{i-1}^{n} w_i ln w_i$$

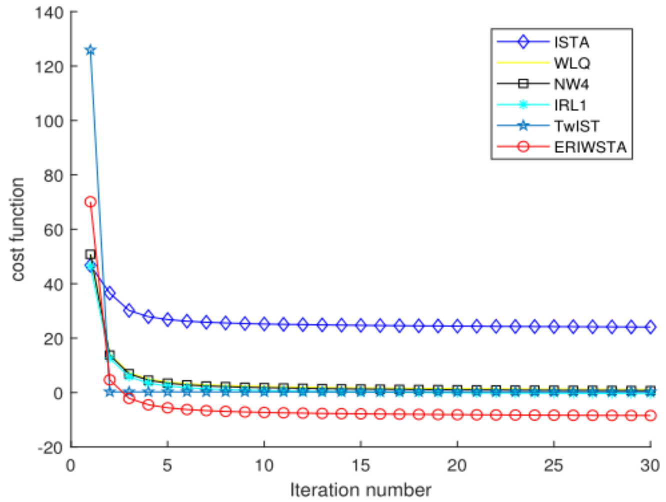

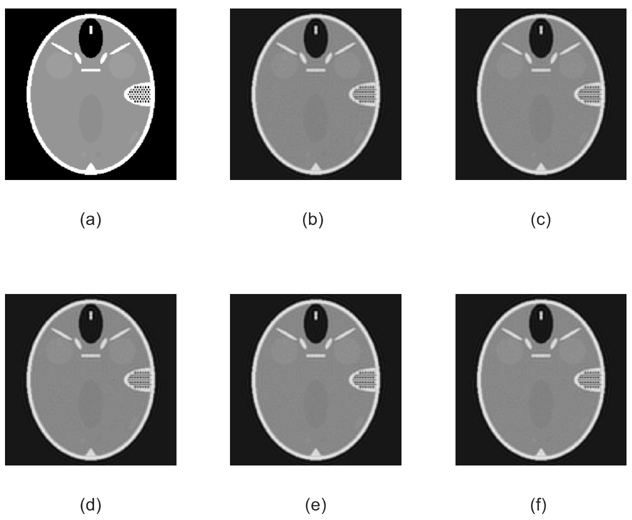

Supplement: S1 Graphical abstract — (PDF) [file pone.0311227.s001.pdf]
